# Supplementary material for: The Interplay of Personality Traits and Psychological Well-Being: Insights from a Study of Italian Undergraduates
Source: Int J Environ Res Public Health. 2025 Jan 21;22(2):132. doi: 10.3390/ijerph22020132 (PMC11855175; doi:10.3390/ijerph22020132)
Supplement: Supplementary file 1 [file ijerph-22-00132-s001.zip › ijerph-3358840-supplementary.pdf]

Supplementary Material

A. Parallel Analysis. A dataset of random numbers, having the same sample size and number of variables of the real dataset is generated by randomly shuffling the numbers within the original dataset. The related eigenvalues are estimated and recorded. This procedure is repeated 1000 times thus obtaining for each component a distribution of 1000 randomly generated eigenvalues. A component of the original dataset is retained only if its eigenvalue exceeds the 95th percentile of the corresponding distribution of randomly generated eigenvalues.

B. Single threshold test for the maximum r-statistic.
We here briefly introduce the basis and rationale of the method: let us assume without loss of generality to have collected a set of variables' loadings on a PCA component. Note that loadings are the correlation coefficients between each variable and the PCA component. As the correlation analysis is applied to multiple variables, a single-threshold procedure is used to assess the significance of each r-value (i.e. loading), tackling with the multiple comparison issue. Let us consider the null-hypothesis of no significant correlation between any variables and the component: under the null-hypothesis, the labeling of each collected variable can be randomly assigned (i.e. for each variable the score of subject A can be assigned to a subject B and so on). Based on this assumption, 10000 random relabeling are made, and for each of them, the r-value related to each single variable-component correlation is estimated. For each relabeling, only the maximum r-value (in absolute value, for two-tailed significance assessment) among simultaneous correlations (i.e. over the variables) is retained. At the end of the procedure, the maximum r-value distribution under the null-hypothesis of no significant correlation between the variables and the PCA component is obtained. The significance of each original r-value (i.e. loading), is then estimated as the ratio between the number of r-values of the null-distribution exceeding the original r-value (in absolute value) and the number of relabeling.

C. Stress, Anxiety, Depression and Insomnia

Table S1. For each component, the related eigenvalue together with the eigenvalue generated using parallel analysis (PA, between brackets), and the variance explained by the component are reported. The retained component (the only one exceeding its corresponding PA eigenvalue), is highlighted in bold letters.

Components	Eigenvalues (PA)	Variance Explained (%)
**1**	**3.51 (1.59)**	**58.55**
2	0.99 (1.33)	16.51
3	0.61 (1.14)	10.13
4	0.35 (1.00)	5.86
5	0.33 (0.88)	5.43
6	0.21 (0.74)	3.51

D. BIG FIVE INVENTORY: Extraversion

Table S2. For each component, the related eigenvalue together with the eigenvalue generated using parallel analysis (PA, between brackets), and the variance explained by the component are reported. The retained component (the only one exceeding its corresponding PA eigenvalue), is highlighted in bold letters.

Components	Eigenvalues (PA)	Variance Explained (%)
**1**	**2.89 (1.62)**	**48.17**
2	1.00 (1.32)	16.67
3	0.78 (1.15)	13.00
4	0.68 (1.01)	11.33
5	0.52 (0.87)	8.67
6	0.13 (0.73)	2.17

E. BIG FIVE INVENTORY: Agreeableness

Table S3 For each component, the related eigenvalue together with the eigenvalue generated using parallel analysis (PA, between brackets), and the variance explained by the component are reported. The retained components (the ones exceeding their corresponding PA eigenvalue), are highlighted in bold letters.

Components	Eigenvalues (PA)	Variance Explained (%)
**1**	**2.84 (1.60)**	**47.33**
2	**1.39 (1.34)**	**23.17**
3	0.71 (1.14)	11.83
4	0.51 (0.99)	8.50
5	0.39 (0.88)	6.50
6	0.15 (0.75)	2.50

Table S4. The loadings of the considered psychometric variables on the extracted components along with their significance are presented. Note that thresholds for significance at p = 0.05 and p = 0.001 are respectively r = 0.31 and r = 0.45 for the first component and r = 0.31 and r = 0.44 for the second one.

Variables	Component 1		Component 2	
	loadings	p-values	loadings	p-values
**BFI-Agreeableness**	-0.046	1.000	0.785	0.001
**LCB-External**	0.748	0.001	-0.083	0.972
**LCB-Internal**	-0.788	0.001	-0.063	0.993
**PRMQ-Total**	0.714	0.001	-0.524	0.001
**MAAS**	-0.125	0.841	0.767	0.001
**PWBF**	0.705	0.001	-0.469	0.001

F. BIG FIVE INVENTORY: Conscentiousness

Table S5. For each component, the related eigenvalue together with the eigenvalue generated using parallel analysis (PA, between brackets), and the variance explained by the component are reported. The retained component (the only one exceeding its corresponding PA eigenvalue), is highlighted in bold letters.

Components	Eigenvalues (PA)	Variance Explained (%)
**1**	**2.90 (1.62)**	**48.33**
2	0.99 (1.33)	16.50

3	0.91 (1.15)	15.17
4	0.64 (1.00)	10.67
5	0.41 (0.87)	6.83
6	0.15 (0.73)	2.50

G. BIG FIVE INVENTORY: Neuroticism

Table S6. For each component, the related eigenvalue together with the eigenvalue generated using parallel analysis (PA, between brackets), and the variance explained by the component are reported. The retained components (the ones exceeding their corresponding PA eigenvalues), are highlighted in bold letters.

Components	Eigenvalues (PA)	Variance Explained (%)
**1**	**2.94 (1.59)**	**49.00**
2	1.04 (1.32)	17.33
3	0.92 (1.14)	15.33
4	0.56 (1.01)	10.67
5	0.40 (0.88)	9.33
6	0.14 (0.74)	2.33

H. BIG FIVE INVENTORY: Openness

Table S7. For each component, the related eigenvalue together with the eigenvalue generated using parallel analysis (PA, between brackets), and the variance explained by the component are reported. The retained component (the ones exceeding their corresponding PA eigenvalues), are highlighted in bold letters.

Components	Eigenvalues (PA)	Variance Explained (%)
**1**	**2.79 (1.60)**	**46.50**
**2**	**1.47 (1.34)**	**24.50**
3	0.81 (1.14)	13.50
4	0.49 (1.00)	8.17
5	0.29 (0.87)	4.83
6	0.15 (0.75)	2.50

Table S8. The loadings of the considered psychometric variables on the two extracted components along with their significance are presented. Note that thresholds for significance at p = 0.05 and p = 0.001 are respectively r = 0.31 and r = 0.45 for the first component and r = 0.32 and r = 0.46 for the second one.

variables	Component 1		Component 2	
	loadings	p-values	loadings	p-values
**BFI-Extraversion**	-0.030	1.000	0.902	0.001
**LCB-External**	0.625	0.001	-0.243	0.221
**LCB-Internal**	-0.380	0.009	0.707	0.993
**PRMQ-Total**	0.904	0.001	-0.136	0.792
**MAAS**	-0.509	0.001	0.189	0.481
**SADI**	0.906	0.001	-0.034	1.000
